# Immature Dendritic Cells Generated from Cryopreserved Human Monocytes Show Impaired Ability to Respond to LPS and to Induce Allogeneic Lymphocyte Proliferation

**DOI:** 10.1371/journal.pone.0071291

**Published:** 2013-07-31

**Authors:** Guilherme Ferreira Silveira, Pryscilla Fanini Wowk, Anália Maria Breckenfeld Machado, Claudia Nunes Duarte dos Santos, Juliano Bordignon

**Affiliations:** 1 Laboratório de Virologia Molecular, Instituto Carlos Chagas, FIOCRUZ, Curitiba, Paraná, Brasil; 2 Laboratório de Regulação da Expressão Gênica, Instituto Carlos Chagas, FIOCRUZ, Curitiba, Paraná, Brasil; 3 Centro de Hematologia e Hemoterapia do Paraná – Divisão de Produção, Curitiba, Paraná, Brasil; University of Bergen, Norway

## Abstract

Dendritic cells play a key role in the immune system, in the sensing of foreign antigens and triggering of an adaptive immune response. Cryopreservation of human monocytes was investigated to understand its effect on differentiation into immature monocyte-derived dendritic cells (imdDCs), the response to inflammatory stimuli and the ability to induce allogeneic lymphocyte proliferation. Cryopreserved (crp)-monocytes were able to differentiate into imdDCs, albeit to a lesser extent than freshly (frh)-obtained monocytes. Furthermore, crp-imdDCs had lower rates of maturation and cytokine/chemokine secretion in response to LPS than frh-imdDCs. Lower expression of Toll-like receptor 4 (at 24 and 48 h) and higher susceptibility to apoptosis in crp-imdDCs than in fresh cells would account for the impaired maturation and cytokine/chemokine secretion observed. A mixed leukocyte reaction showed that lymphocyte proliferation was lower with crp-imdDCs than with frh-imdDCs. These findings suggested that the source of monocytes used to generate human imdDCs could influence the accuracy of results observed in studies of the immune response to pathogens, lymphocyte activation, vaccination and antigen sensing. It is not always possible to work with freshly isolated monocytes but the possible effects of freezing/thawing on the biology and responsiveness of imdDCs should be taken into account.

## Introduction

Dendritic cells (DC), which were first described in 1973 [1], are a major component of the immune system. They are involved in the sensing of foreign antigens and the processing and presenting of antigens to lymphocytes. DCs are the main antigen-presenting cells (APC) in the immune system, bridging the gap between adaptive and innate immune responses [2]. For these reasons, DCs are often the cells chosen to study the pathogenesis of diseases caused by infectious agents, vaccine responses, cancers and autoimmune diseases [3]–[8]. Additional functions of DCs, such as the control of lymphocyte migration to lymph nodes [9], show their wide relevance to immunology. Dendritic cell-based immunotherapy against cancer has attracted considerable attention in recent years and has increased the importance of this cell type in medicine and basic science [7].

Blood monocytes (CD14^+^) are the major source of human DCs *in vitro*. Their differentiation into DCs (immature monocyte-derived dendritic cells or imdDCs) can be induced *in vitro* by interleukin-4 (IL-4) and granulocyte/macrophage colony-stimulating factor (GM-CSF) [10]. Cryopreservation of CD14^+^ cells is important when studying rare samples, for analyzing cells collected in areas without laboratory facilities and to avoid multiple sampling of the same patient. Methods for isolating and cryopreserving CD14^+^ cells from human blood have been described and standardized [7], [11]. However, there have been conflicting reports concerning the effects of cryopreservation on monocyte differentiation, the response to antigens and the allogeneic stimulation of T cells [11]–[14].

Given the crucial role of DCs in the immune system regulation and their response to pathogens, evaluating the effect of cryopreservation on these cells is relevant. The freezing and thawing of human monocytes was investigated to understand if it had any effect on cell differentiation, the response to inflammatory stimuli, cell survival or lymphocyte proliferation. Cryopreserved (crp)-monocytes differentiated into immature-mdDCs (crp-imdDCs) at a lower frequency and displayed lower rates of maturation and cytokine secretion in response to LPS than freshly obtained (frh)-imdDCs. The reduced differentiation of crp-CD14^+^ to imdDCs could be explained by the lower expression of GM-CSF and IL-4 receptors on crp-CD11c^+^ during differentiation. The impaired response of crp-imdDCs to LPS may be explained by the lower TLR4 expression and increased susceptibility to apoptosis. Crp-imdDCs were also less able to induce allogeneic lymphocyte proliferation than frh-imdDCs.

## Materials and Methods

### Purification of CD14^+^ Cells, Cryopreservation and imdDC Differentiation

This project was authorized by the FIOCRUZ Research Ethics Committee (#514/09). Peripheral blood was obtained from healthy donors who gave written informed consent to participate. CD14^+^ cells were purified and induced to differentiate into imdDCs as previously described [15].

CD14^+^ cells were cryopreserved (crp-CD14^+^) in inactivated fetal bovine serum (iFBS; Gibco-BRL, Grand Island, NY) containing 10% DMSO (Sigma-Aldrich, St. Louis, MO) as previously described [12], [16]. CD14^+^ cells were thawed by incubation for 1 to 2 min at 37°C in a water bath, and washed with iFBS by centrifugation at 300×*g* for 10 min. Fresh or cryopreserved CD14^+^ cells (5×10^5^ viable cells due to Trypan Blue exclusion assay) were incubated in RPMI medium (Lonza, Walkersville, USA) supplemented with 10% iFBS, 100 IU/mL of penicillin, 100 µg/mL of streptomycin, 2 mM of L-Glutamine, 2.5 µg/mL of amphotericin (Gibco-BRL, Grand Island, NY), 100 ng/mL IL-4 and 50 ng/mL GM-CSF (PeproTech, Rocky Hill, NJ) for 6 to 7 days, as previously described [15]. The expression of imdDCs markers (CD11c and HLA-DR) and CD14 (PE, PercP and FITC conjugated antibodies, respectively; BD Pharmigen™, USA) were evaluated in frh- and crp-imdDCs by flow cytometry in a FACSAria II (Becton & Dickinson, San Jose, CA), as described by Silveira *et al.* (2011). Moreover, CD116 (GM-CSF receptor) and CD124 (IL-4 receptor) (FITC and PE conjugated antibodies, respectively; BD Pharmigen™, USA) expression were evaluated at 0, 4 and 7 days after induced differentiation. Isotype-matching antibodies were used as negative controls (eBioscience, San Diego, CA, USA).

### Response to Inflammatory Stimuli

After viability determination (Trypan blue exclusion assay), 5×10^5^ frh- and crp-imdDCs were stimulated with the bacterial endotoxin lipopolysaccharide (LPS from *Escherichia coli*; Ingenex, San Diego, CA, USA), at a concentration of 1 µg/mL in RPMI supplemented medium (without IL-4 and GM-CSF) for 6, 24 and 48 h. Cells were harvested, centrifuged and the supernatants stored at −80°C for cytokine/chemokine determination. Cells were analyzed for the expression of CD40, CD80, CD83 and CD86 costimulatory markers (APC, PE-Cy7, FITC and PE conjugated antibodies, respectively; BD Pharmigen™, USA) and TLR4 expression (biotin conjugated antibody plus streptavidin-FITC) by flow cytometry in a FACSAria II (Becton & Dickinson, San Jose, CA). Chemokine (IP-10, MCP-1 and RANTES) and cytokine (TNF-α and IL-6) levels were determined in cell-free supernatants with the CBA Flex Set Kit (Becton & Dickinson, San Jose, CA).

### Apoptosis

Annexin V and propidium iodide (PI) or 7-AAD (eBioscience, San Diego, AS, USA) were used to determine the percentage of apoptotic cells. The frh- and crp-imdDCs were labeled according to the manufacturer’s recommendations. Briefly, crp- and frh-imdDCs were treated with LPS, harvested, washed with wash buffer and incubated in 45 µL of binding buffer with 5 µL of Annexin V for 15 min. Cells were washed again in buffer, recovered in 300 µL of PBS (Lonza, Walkersville, USA) and 5 µL of PI or 7-AAD, and analyzed on a FACSAria II (Becton & Dickinson, San Jose, CA).

### Mixed Leukocyte Reaction

DCs and lymphocytes co-cultures (allogeneic or autologous) were performed to investigate the ability of frh- and crp-imdDCs to stimulate lymphocyte proliferation. After monocyte-DC differentiation, new samples of lymphocytes (CD14^+^ depleted PBMC) were obtained from the same donors and stained with CellTracker™ Green CMFDA (Invitrogen, Carlsbad, USA) according to the manufacturer’s instructions. Cultures were set up with a 1∶10 ratio of frh- or crp-imdDCs to autologous or allogeneic freshly obtained lymphocytes, in a 6-well plate with RPMI supplemented medium (without IL-4 and GM-CSF). The proliferation rate was analyzed after five days using a FACSAria II (Becton & Dickinson, San Jose, CA).

### Statistical Analysis

Statistical differences between the groups were analyzed using Student’s *t* test, one- or two-way ANOVA followed by a Bonferroni post-test, in GraphPad Prism 3.0 (GraphPad Software, SanDiego, CA). A *p* value of 0.05 or less was considered statistically significant.

## Results

### Crp-CD14^+^ Cells Differentiated at Lower Levels than frh-CD14^+^ Cells

**Figure 1:**
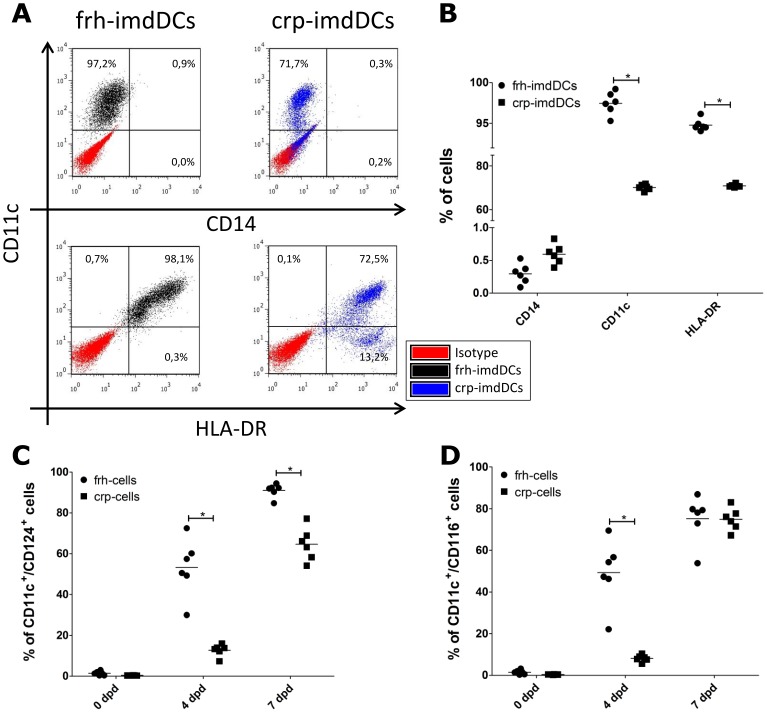
Phenotypic analyses of frh- and crp-imdDCs. Frh-imdDCs (black), crp-imdDCs (blue) and isotype-matched controls (red) were analyzed for the expression of CD11c/CD14 and CD11c/HLA-DR. (A) Representative dot blot analyses for the expression of CD11c/CD14 and CD11c/HLA-DR from one blood donor. (B) Data from six independent cultures of frh- (circles) and crp-imdDCs (squares). Data was analyzed by Student’s *t* test and the values shown are the means ± SDs of six individual donors. (C and D) Expression of IL-4 (CD124) and GM-CSF (CD116) receptors on CD11c^+^ cells during monocytes differentiation to imdDCs. Frh-monocytes (circles) and crp-monocytes (squares) were induced to differentiate to imdDCs and growth factors receptors were analyzed at 0, 4 and 7 days post-differentiation (dpd). Data was analyzed using one-way ANOVA followed by a Bonferroni test; values represent means ± SDs of six individual donors. *p≤0.05, frh: freshly obtained, crp: cryopreserved, imdDCs: immature monocyte derived dendritic cells.

Immature monocyte-derived dendritic cells express high levels of CD11c and HLA-DR, but lose their CD14 expression during differentiation [10], [17]. To investigate whether CD14^+^ cells cryopreservation before differentiation affected the phenotype of imdDCs, we evaluated the expression of markers involved in DC differentiation (Figure 1 A). Expression of CD11c and HLA-DR was lower in imdDCs derived from crp-CD14^+^ than in those derived from frh-CD14^+^ cells. Almost no CD14 expression was observed in either type of differentiated cell (Figure 1 B). Lower expression of IL-4 receptor (CD124) at 4 and 7 days post-differentiation (dpd) and GM-CSF receptor (CD116) 4 dpd was associated with crp-CD11c^+^ than with fresh-cells during the differentiation of monocytes to imdDCs (Figure 1 C and D). Both crp- and frh-CD14^+^ expressed IL-4 and GM-CSF receptors at similar levels before differentiation to imdDCs began (Figure S1).

Viable CD14^+^ cells were separated (by cell sorting) from crp-CD14^+^ cells to verify if the impaired differentiation of crp-CD14^+^ could be due to a tolerogenic profile induced by apoptotic cells in thawed cultures during differentiation (30% in crp-monocytes; Figure S2 C and D). The exclusion of apoptotic monocytes from the culture did not enhance differentiation efficiency or imdDC phenotype (Figure S2 A and B).

### Response of crp-imdDCs to LPS

**Figure 2:**
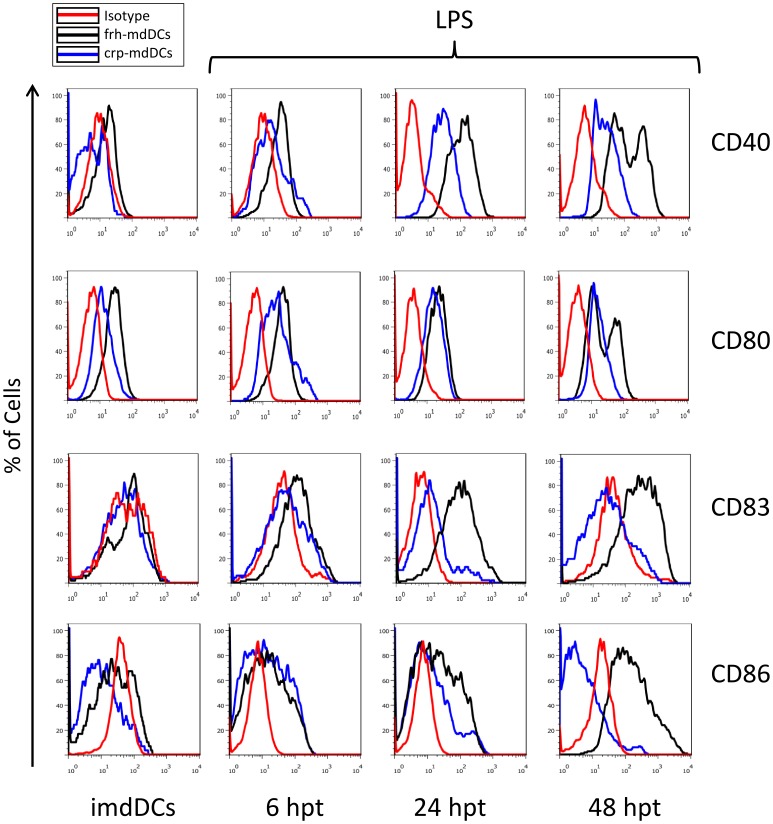
Maturation of frh- and crp-imdDCs after stimulation with LPS. Expression of CD40, CD80, CD83 and CD86 on frh- and crp-imdDCs after treatment with LPS (1 µg/mL) at 0 (imdDCs), 6, 24 and 48 h. Histogram of one individual donor (representative of six). frh: freshly obtained, crp: cryopreserved, imdDCs: immature monocyte derived dendritic cells.

One of the key features of DCs is their ability to recognize and respond to antigens [2], [18], [19]. Having shown that crp-CD14^+^ cells differentiate into imdDCs, the ability of these cells to respond to an inflammatory stimulus (LPS, a TLR4 ligand that induces the expression of mdDC maturation markers and cytokines/chemokines production) was analyzed [20], [21]. Crp- and frh-imdDCs (5×10^5^ viable cells based on Trypan Blue exclusion assay) were treated with LPS and analyzed for the expression of activation markers (CD40, CD80, CD83 and CD86) at 0, 6, 24 and 48 hours post-treatment (hpt) [22]. Crp-imdDCs expressed lower levels of all four activation markers than frh-imdDCs at 48 hpt (Figure 2 and Figure S3).

**Figure 3:**
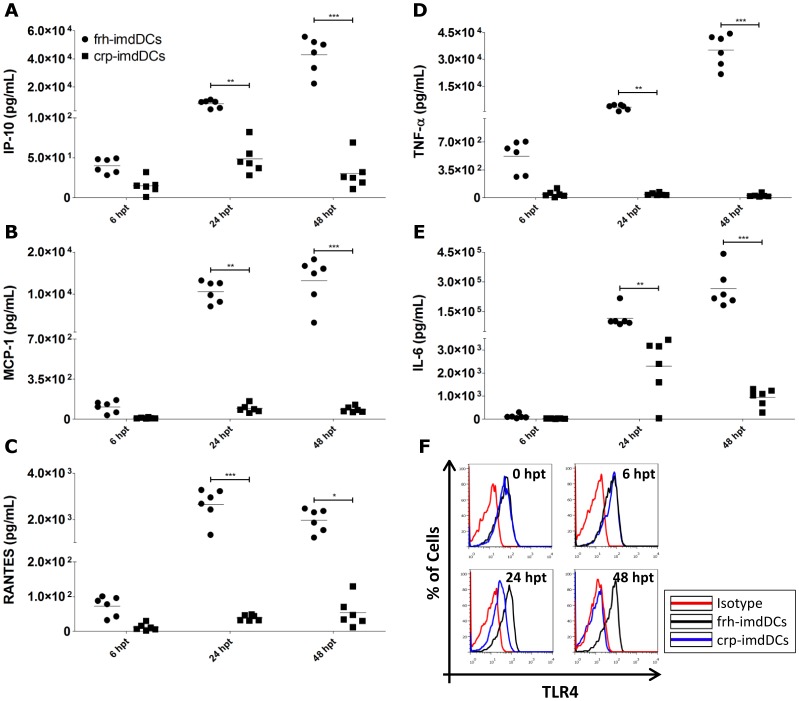
Chemokine/cytokine secretion and TLR4 expression by frh- and crp-imdDCs after stimulation with LPS. After 6, 24 and 48 h of LPS stimulation (1 µg/mL), cell culture supernatants of frh- (circles) and crp-imdDCs (squares) were analyzed with the CBA Flex set kit for IP-10 (A), MCP-1 (B), RANTES (C), TNF-α (D) and IL-6 (E); detection limit for all cytokines/chemokines: 10 pg/mL. Data was analyzed by Student’s *t* test and the values shown are the means ± SDs of six individual donors. *p≤0.05, **p≤0.01, ***p≤0.001. (F) Histograms showing TLR4 expression on the surface of frh- (black) and crp-imdDCs (blue) treated with LPS (red for isotype-matched control). The histogram uses data from one representative donor out of six independent ones. frh: freshly obtained, crp: cryopreserved, imdDCs: immature monocyte derived dendritic cells.

The production of inflammatory cytokines and chemokines was also assessed. Crp-imdDCs cells produced smaller amounts (between 10 and 100 times lower levels) of IP-10, MCP-1, RANTES, TNF-α and IL-6 than frh-imdDCs at 24 and 48 hpt (Figure 3 A–E). The lower levels of cytokine/chemokine secretion by crp-imdDCs may be due to the lower levels of TLR4 expression on cell surfaces at the same time points (Figure 3 F and Figure S4 A). Similarly, a lower expression of CD11c, a known LPS receptor on leukocytes [23], was observed in crp-imdDCs than in frh-cells (Figure S4 B and C).

### Crp-imdDCs are More Susceptible to Apoptosis than frh-imdDCs

**Figure 4:**
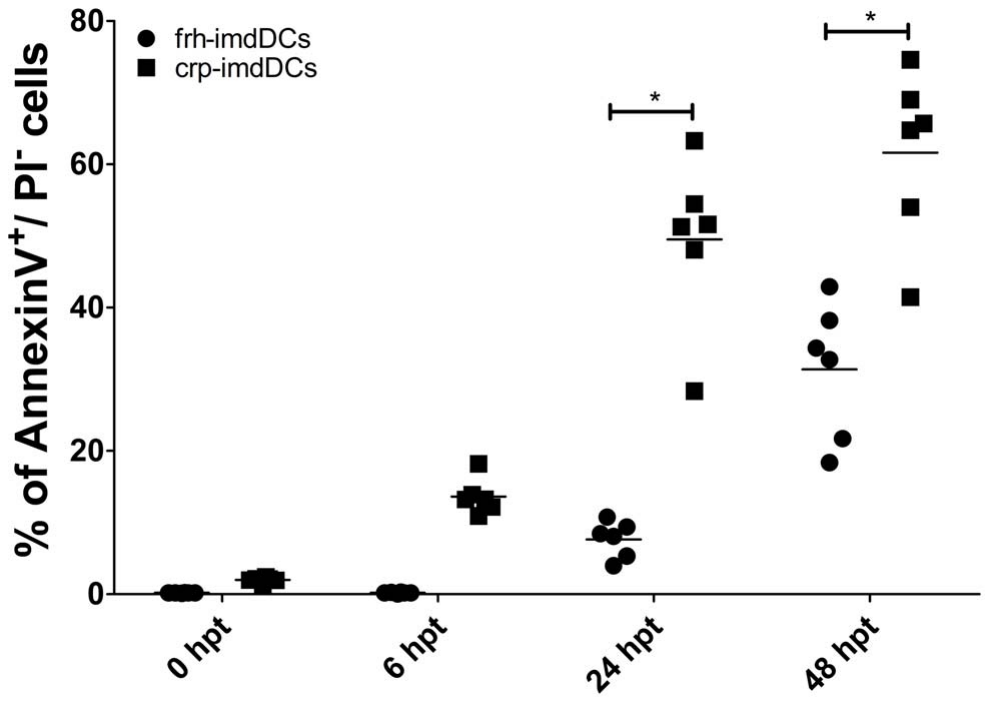
Apoptosis of frh- and crp-imdDCs. Analysis of apoptosis (Annexin V^+^/PI^-^) in frh- (circles) and crp-imdDCs (squares) at 0, 6, 24 and 48 h. Data was analyzed by Student’s *t* test and the values shown are the means ± SDs of six individual donors. *p≤0.05.

Apoptosis, a programmed cell death, is a fundamental control mechanism of the immune response [24]. Previous studies have shown that imdDCs are susceptible to apoptosis after differentiation in the absence of specific stimuli and can represent 10 to 20% of cells in culture after 48 h [15]. Staining for annexin V and PI was assessed in crp- and frh-imDCs to evaluate if the lower response of crp-imdDCs to LPS could also be accounted for by a higher susceptibility to apoptosis. The percentage of annexin V^+^/PI^-^ cells was higher in cpr-imdDCs than in frh-imdDCs at 24 and 48 h (Figure 4). This higher susceptibility to apoptosis in crp-imdDCs may explain the lower response to LPS. Additionally, dying cells were found to have lower levels of CD11c, CD40 and CD80 expression (Figure S5).

### Crp-imdDCs are Less Able to Induce Lymphocyte Proliferation in a Mixed Leukocyte Reaction

**Figure 5:**
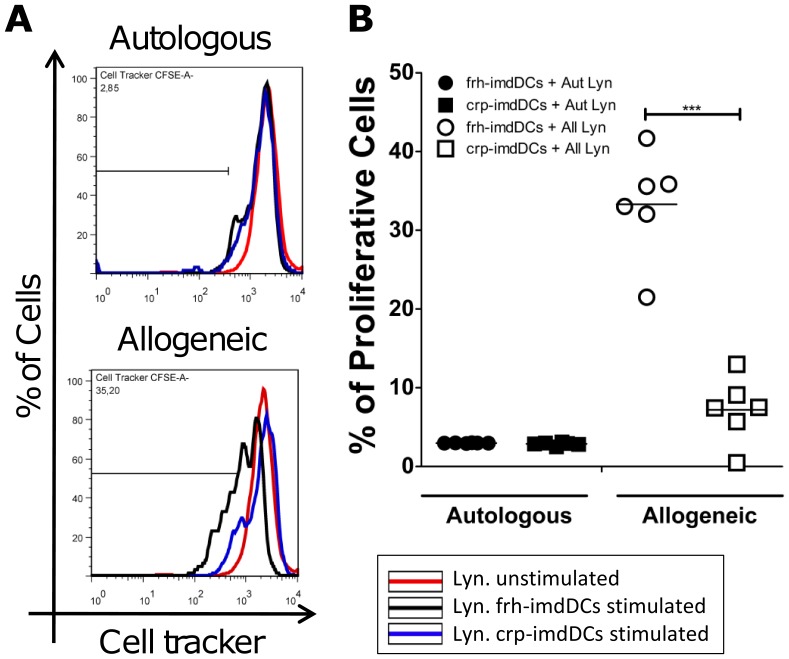
Cryopreservation interferes with the allogeneic lymphoproliferative response. (A) Representative histogram of autologous and allogeneic lymphocyte proliferation of one donor (frh-imdDCs (black), crp-imdDCs (blue) and Lyn-stained lymphocytes without DC stimulation (red)). (B) Frh- (circles) and crp-imdDCs (squares) were co-cultured with autologous (negative control) or allogeneic lymphocytes stained with CellTracker™ Green CMFDA in a 1∶10 ratio of imdDCs:lymphocytes. After five days of co-culture lymphocytes proliferation was analyzed by flow cytometry. Data was analyzed by Student’s *t* test and the values shown are the means ± SDs of six individual donors. ***p≤0.001. frh: freshly obtained, crp: cryopreserved, imdDCs: immature monocyte derived dendritic cells, Lyn: stained lymphocytes without DC stimulation, Aut lyn: autologous lymphocytes, All lyn: allogeneic lymphocytes.

Co-cultures were performed with allogeneic lymphocytes to evaluate DC function as a specialized antigen-presenting cell. Autologous lymphocytes were used to observe lymphocyte basal proliferation. Figure 5 shows that co-culture of frh- or crp-imdDCs with autologous lymphocytes resulted in an absence of T-cell proliferation, as expected. However, co-culture of frh-imdDCs with allogeneic lymphocyte resulted in approximately three times more lymphocyte proliferation than co-culture with crp-imdDCs, suggesting that cryopreservation reduces the functionality of dendritic cells.

## Discussion

Dendritic cells are the most potent antigen-presenting cells and are responsible for crosstalk between the innate and adaptive immune responses [2], [18], [19]. Since their initial description in 1973 [1], the known role of DCs in both basic science and medicine has increased to include allergy control [3], viral infections [4], [5], transplantation [6], autoimmune diseases [25], cancer [8] and immune regulation [9]. The importance of DCs in the immunological process was acknowledged by the 2011 Nobel Prize for Physiology or Medicine award to Ralph M. Steinman who first observed them (http://www.nobelprize.org/nobel_prizes/medicine/laureates/2011/). One of the most common protocols for human DC differentiation uses CD14^+^ monocytes from peripheral blood [10]. The cryopreservation of CD14^+^ cells provides a source of imdDCs for later use and is an important procedure in studies based on these cells [7], [13], [26]. However, the effects of cryopreservation on CD14^+^ cells remain unclear. The freeze/thaw cycle procedure may modify the differentiation of DCs and the subsequent response of these cells to inflammatory stimuli or pathogens [12].

In this study, crp-CD14^+^ cells were found to be able to differentiate into imdDCs but did so less efficiently than frh-CD14^+^ cells (Figure 1). This suggests that the freeze/thaw procedure does indeed modify these cells. The observed differences in differentiation ability between the two cell types may be due to the presence of fewer IL-4/GM-CSF receptors on crp-CD11c^+^ during differentiation (Figure 1 C and D). Consistent with this notion, levels of CD11c and HLA-DR expression were lower on the surface of crp-imdDCs than on the surface of frh-imdDCs (Figure 1 A and B). Furthermore, cryopreservation of monocytes induced approximately 30% of cells to death (Annexin V^+^/7-AAD^-^, Figure S2 C and D) and could have influenced differentiation to imdDCs. The exclusion of apoptotic monocytes after thawing (by cell sorting) resulted in similar differentiation to imdDCs than in unsorted crp-CD14^+^ (Figure S2 A and B). This suggests that the presence of apoptotic cells in the culture after thawing does not interfere with the differentiation of monocytes to imdDCs.

Makino and Baba (1997) demonstrated that mdDCs generated from frozen PBMCs showed similar levels of CD80, CD86, HLA-DQ and HLA-DR to mdDCs generated from fresh PBMCs. Ghanekar *et al.* (2007) showed that cryopreserved monocytes from cancer patients could be differentiated fully into mature DCs with a phenotype and function similar to those of DCs derived from the cells of healthy donors. These different results may be explained by the procedures employed for CD14^+^ cell isolation and cryopreservation. Makino and Baba, (1997) and Ghanekar *et al.* (2007) applied adherence to plastic for cell separation whereas magnetic sorting was used in this study. Moreover, DMSO and rIL-4/rGM-CSF concentration (used for freezing cells and to differentiate monocytes into imdDCs, respectively), varied between studies and could help to explain the distinct results. This study used cells from healthy donors whereas Ghanekar *et al.* (2007) used monocytes from cancer patients. Furthermore, it analyzed a larger number of donors (six) and different parameters than in other studies. There is no consensus on what should be the gold standard protocol for cryopreserve monocytes. Cell type (PBMCs or CD14^+^), cell source (healthy or non-healthy donors), DMSO concentration, medium, cell number and other factors could therefore also contribute to observed differences.

DC maturation is a critical step in the presentation of antigens to T cells and the initiation of an adaptive immune response [27]. The treatment of DCs with LPS can be used to demonstrate cell activation, which can be characterized by the pattern of surface markers [20], [21]. Crp-imdDCs expressed lower levels of activation markers (CD40, CD80, CD83 and CD86) than frh-imdDCs at 48 hpt with LPS (Figure 2 and Figure S3). This suggests that imdDCs generated from frozen monocytes have an impaired response to inflammatory stimuli, in contrast to the results reported by Hayden *et al*. (2009).

Cytokines and chemokines are important mediators secreted by DCs in response to pathogens triggering an immune response [2], [19]. The stimulation of imdDCs with LPS induces these cells to secrete inflammatory cytokines such as IL-12p40, IL-1β, TNF-α and IL-6 [20], [21], [28]. Crp-imdDCs were shown to secrete smaller amounts of inflammatory cytokines/chemokines than frh-imdDCs (Figure 3 A–E) after stimulation with LPS. These findings are consistent with those of Meijerink *et al*. (2011), who showed that crp-imdDCs secreted smaller amounts of TNF-α, IL-1β and IL-12p70 after stimulation with lipoteichoic acid, LPS and bacteria. The response of imdDCs to LPS depends on the expression of TLR4 [29] and CD11c [23] at the cell surface and so levels of TLR4 and CD11c expression were determined in frh- and crp-imdDCs. Fewer TLR4 and CD11c molecules were found on the surface of crp-imdDCs than on frh-imdDCs (Figure 3 F and S4), potentially explaining the lower response of crp-imdDCs to LPS stimulation. Low expression of cellular receptors has also been demonstrated for monocytes and lymphocytes obtained from cryopreserved-PBMCs. These cells expressed lower levels of PD-1 receptor and PD-L1 ligand on their surface, resulting in a weaker lymphoproliferative response to PD-1/PD-L1 blockade [30].

Apoptosis plays an important role in the control and regulation of the immune response [31], [32] and DCs have been shown to have a high turnover rate [24]. Frh-imdDCs undergo apoptosis in a time-dependent manner in the absence of specific stimuli [15]. In this study, there were a larger number of apoptotic cells (Annexin V^+^/PI^-^) in crp-imdDCs than frh-imdDCs, confirming the greater susceptibility of imdDCs derived from crp-CD14^+^ cells to apoptosis (Figure 4). Lower levels of TLR4/CD11c expression and higher susceptibility to apoptosis in crp-imdDCs could account for their lower response to LPS than frh-imdDCs.

A mixed leukocyte reaction experiment was performed to evaluate if the differences in cell phenotype and responsiveness impaired the antigen presentation ability of DCs [33]. As expected, both imdDC types (cryopreserved or freshly obtained) were unable to stimulate lymphocyte proliferation when co-cultured with autologous lymphocytes, as autologous lymphocytes are not able to recognize self-antigens presented by imdDC. However, when allogeneic lymphocytes were stimulated by imdDC, frh-imdDCs induced more lymphocyte proliferation than crp-imdDCs (Figure 5). This suggests that cryopreservation of monocytes impairs the ability of crp-imdDCs to present antigens to T-cells.

Taken together, these results demonstrate that cryopreserved monocytes generate dendritic cells less efficiently than frh-CD14^+^ cells and that the imdDCs generated from crp-CD14^+^ cells display impaired maturation and cytokine/chemokine secretion after exposure to inflammatory stimuli (LPS). The lower responsiveness of crp-imdDCs to LPS seems to be due to lower levels of TLR4/CD11c expression at the cell surface and a higher susceptibility of crp-imdDCs to apoptosis. Allogeneic proliferation was impaired in crp-imdDCs when compared to proliferation in frh-imdDCs (as demonstrated by the mixed leukocyte reaction). The source of the monocytes from which DCs differentiate may therefore affect imdDC phenotype, maturation, cytokine/chemokine production, survival and DC function with repercussions for studies on host-pathogen interactions, vaccine response, allogeneic T-cell stimulation and immune regulation.

## Supporting Information

Figure S1 **Expression of GM-CSF and IL-4 receptors on CD14^+^.** Expression of IL-4 (CD124; A) and GM-CSF (CD116; B) receptors on CD14^+^ cells during monocytes differentiation to imdDCs. Fresh (circles) and crp-monocytes (squares) were induced to differentiate into imdDCs using IL-4 and GM-CSF, and receptors for growth factors were analyzed at 0, 4 and 7 days post-differentiation (dpd). Data was analyzed using one-way ANOVA followed by a Bonferroni test; values represent means ± SDs of the results of six individual donors. *p≤0.05.
(TIF)

Figure S2 **Differentiation of crp-monocyte into imdDCs with sorting of viable cells after thawing.** (A) Expression of imdDCs markers (CD11c and HLA-DR) on differentiated crp-sorted and unsorted monocytes from one donor. (B) Percentage of cells expressing CD14, CD11c and HLA-DR after differentiation to imdDCs in crp-sorted and unsorted monocytes from six different donors. (C) Exclusion of dying cells (Annexin V^+^/7-AAD^−^) in monocytes cultures after thawing using cell sorting from one representative donor. (D) Percentage of viable cells (Annexin V^−/^7-AAD^−^) from six different donors in sorted and unsorted monocytes after thawing. Data was analyzed using two-way ANOVA followed by a Bonferroni test; values represent means ± SDs of the results from six different experiments.
(TIF)

Figure S3 **Mean fluorescence intensity of activation markers on imdDCs generated from crp- and frh-monocytes after LPS stimulation.** Frh- and crp-CD14^+^ were induced to differentiate on imdDCs with IL-4 and GM-CSF for 7 days and the expression of CD40, CD80, CD83 and CD86 were analyzed. After monocyte differentiation, cells were stimulated with 1 µg/mL of LPS and cell activation markers were analyzed at 0, 6, 24 and 48 hours post-treatment (hpt). Data was analyzed using two-way ANOVA followed by a Bonferroni test; values represent means ± SDs of the results from six different experiments. *p≤0.05.
(TIF)

Figure S4 **TLR4 and CD11c expression on crp- and frh-imdDCs after LPS stimulation.** TLR4 (A) and CD11c (B) expression was observed at 0, 6, 24 and 48 hours post-treatment (hpt) using 1 µg/mL of LPS. Data was analyzed using two-way ANOVA followed by a Bonferroni test; values represent means ± SDs of the results from six different experiments. *p≤0.05, **p≤0.01. Histogram: CD11c expression on crp- and frh-imdDC cell surfaces after stimulation with LPS. Data from one representative donor out of six (C).
(TIF)

Figure S5 **Expression of CD11c and activation markers on the cell surfaces of live and dying cells.** Expression of CD40, CD80, CD86 and CD11c on live (Annexin V^−/^7-AAD^−^) and apoptotic (Annexin V^+^/7-AAD^−^) frh-imdDCs after treatment with LPS (1 µg/mL) for 48 h. Histogram showing data from one representative donor out of six independent ones.
(TIF)
