# Supplementary figures and images for: Immature Dendritic Cells Generated from Cryopreserved Human Monocytes Show Impaired Ability to Respond to LPS and to Induce Allogeneic Lymphocyte Proliferation
Source: PLoS One. 2013 Jul 31;8(7):e71291. doi: 10.1371/journal.pone.0071291 (PMC3729849; doi:10.1371/journal.pone.0071291)

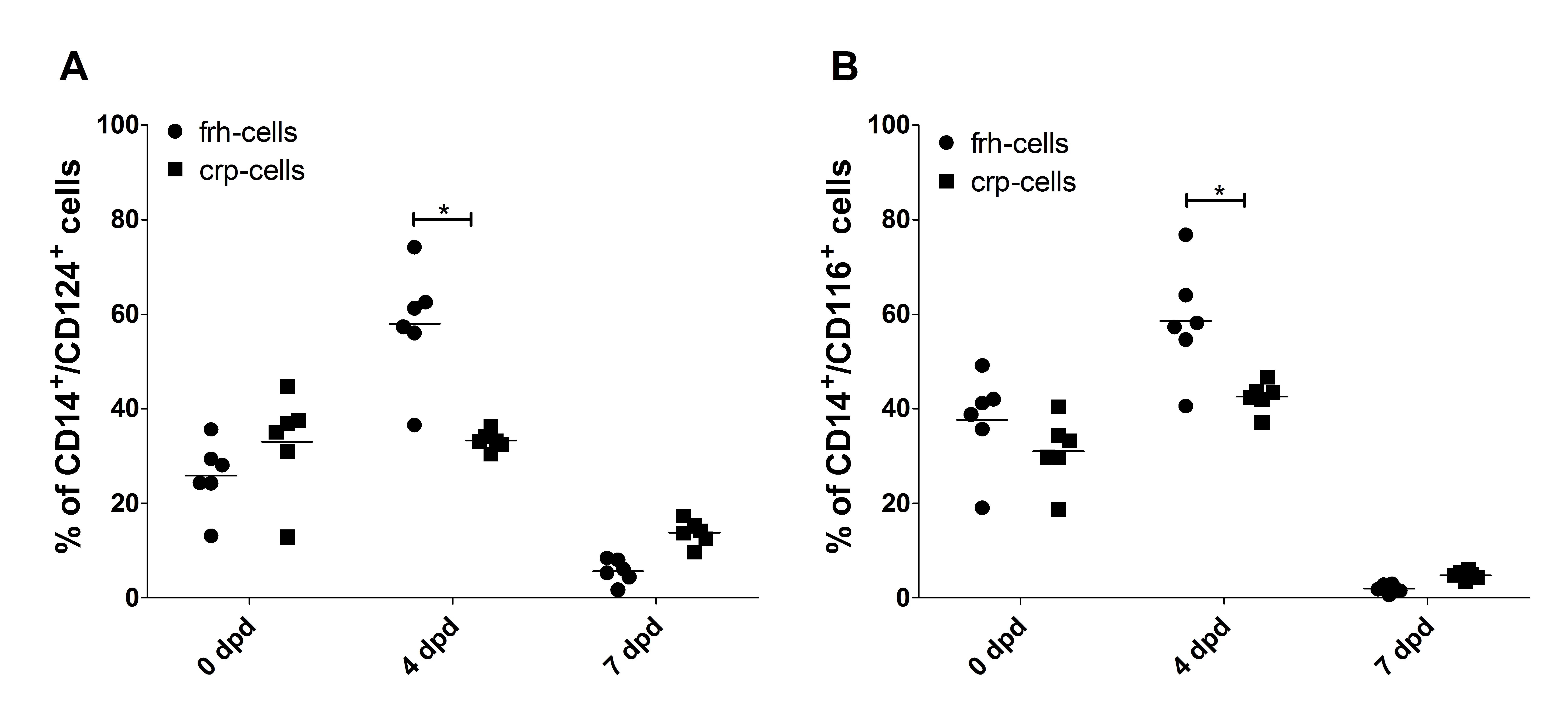

Supplement: Figure S1 — Expression of GM-CSF and IL-4 receptors on CD14+. Expression of IL-4 (CD124; A) and GM-CSF (CD116; B) receptors on CD14+ cells during monocytes differentiation to imdDCs. Fresh (circles) and crp-monocytes (squares) were induced to differentiate into imdDCs using IL-4 and GM-CSF, and receptors for growth factors were analyzed at 0, 4 and 7 days post-differentiation (dpd). Data was analyzed using one-way ANOVA followed by a Bonferroni test; values represent means ± SDs of the results of six individual donors. *p≤0.05. (TIF) [file pone.0071291.s001.tif]

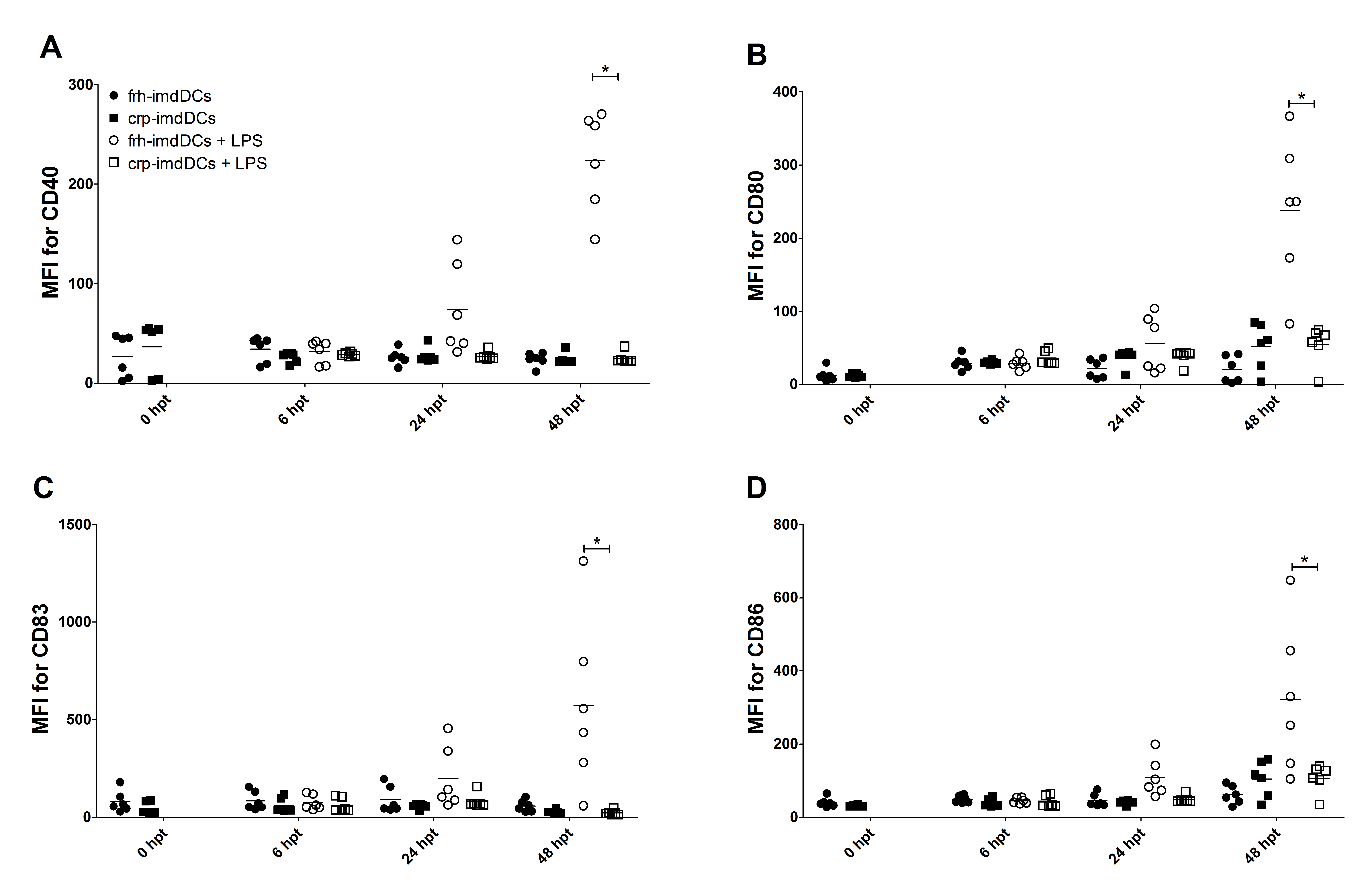

Supplement: Figure S3 — Mean fluorescence intensity of activation markers on imdDCs generated from crp- and frh-monocytes after LPS stimulation. Frh- and crp-CD14+ were induced to differentiate on imdDCs with IL-4 and GM-CSF for 7 days and the expression of CD40, CD80, CD83 and CD86 were analyzed. After monocyte differentiation, cells were stimulated with 1 µg/mL of LPS and cell activation markers were analyzed at 0, 6, 24 and 48 hours post-treatment (hpt). Data was analyzed using two-way ANOVA followed by a Bonferroni test; values represent means ± SDs of the results from six different experiments. *p≤0.05. (TIF) [file pone.0071291.s003.tif]

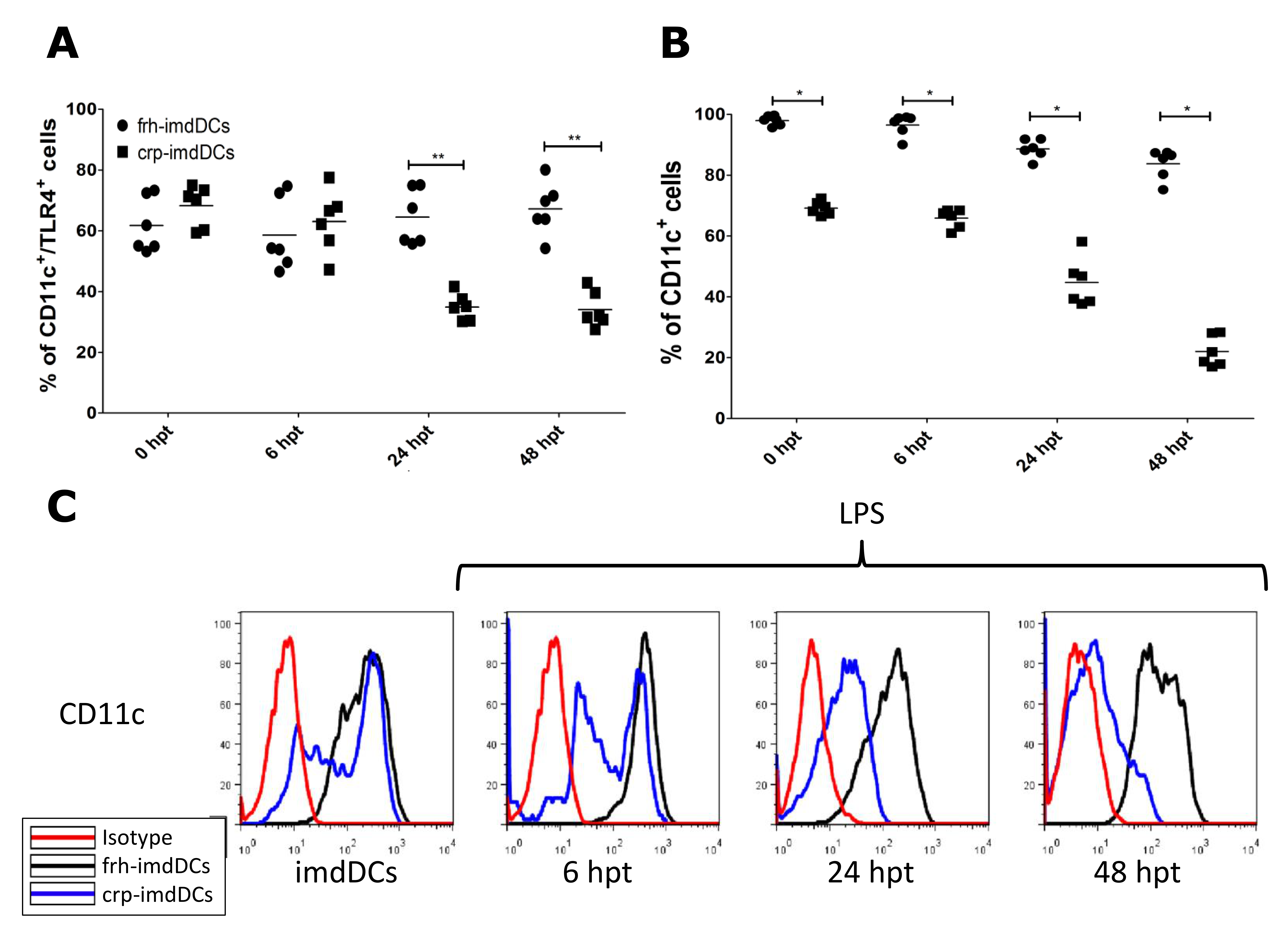

Supplement: Figure S4 — TLR4 and CD11c expression on crp- and frh-imdDCs after LPS stimulation. TLR4 (A) and CD11c (B) expression was observed at 0, 6, 24 and 48 hours post-treatment (hpt) using 1 µg/mL of LPS. Data was analyzed using two-way ANOVA followed by a Bonferroni test; values represent means ± SDs of the results from six different experiments. *p≤0.05, **p≤0.01. Histogram: CD11c expression on crp- and frh-imdDC cell surfaces after stimulation with LPS. Data from one representative donor out of six (C). (TIF) [file pone.0071291.s004.tif]

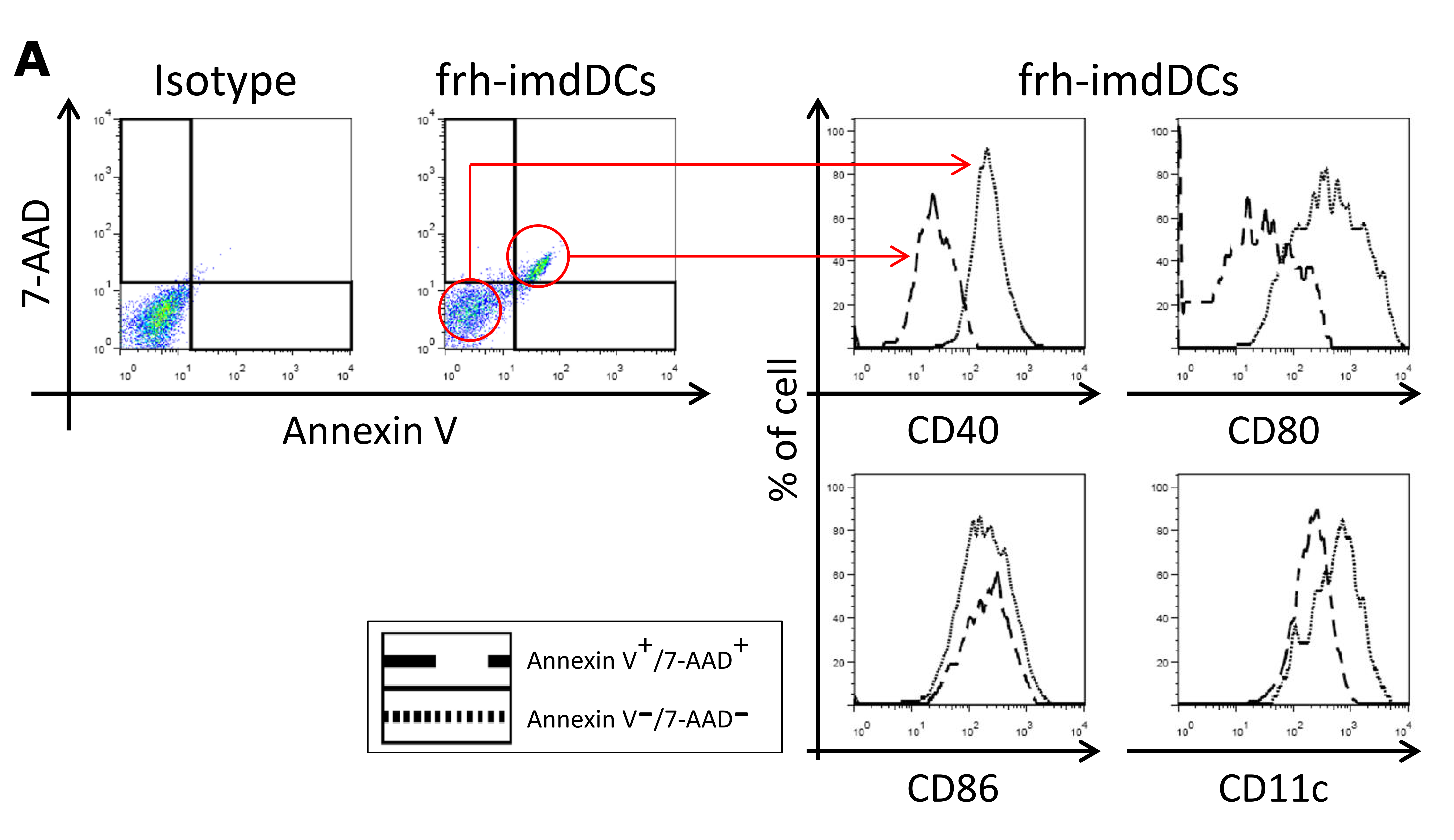

Supplement: Figure S5 — Expression of CD11c and activation markers on the cell surfaces of live and dying cells. Expression of CD40, CD80, CD86 and CD11c on live (Annexin V−/7-AAD−) and apoptotic (Annexin V+/7-AAD−) frh-imdDCs after treatment with LPS (1 µg/mL) for 48 h. Histogram showing data from one representative donor out of six independent ones. (TIF) [file pone.0071291.s005.tif]
